# Synthesis and Biological Evaluation of New Imidazolium and Piperazinium Salts of Pyropheophorbide-*a* for Photodynamic Cancer Therapy

**DOI:** 10.3390/ijms9081407

**Published:** 2008-08-13

**Authors:** Gerelt-Ireedui Sengee, Narangerel Badraa, Young Key Shim

**Affiliations:** PDT Research Institute, School of Nano Engineering, Inje University, Gimhae 621-749, Korea

**Keywords:** piperazinium salts, imidazolium salts, pyropheophorbide-a, photodynamic therapy

## Abstract

We have designed imidazolium and piperazinium salts of pyropheophorbide-*a* in order to develop effective photosensitizers which have good solubility in polar and non polar media and to reveal the possible influences of the piperazine and imidazole moieties on the biological activities of pyropheophorbide-*a.* The phototoxicity of those pyropheophorbide-*a* salts against A549 cells was studied *in vitro* and compared with that of pyropheophorbide-*a*. The result showed that complexing piperazine and imidazole into pyropheophorbide-*a* decreases its dark toxicity without greatly decreasing phototoxicity and, enhances its phototoxicity without greatly increasing dark toxicity, respectively. This work not only describes novel amphiphilic salt complexes of pyropheophobide-*a* which retain the biological activities of the parent compound pyropheophorbide-*a* and could be effective candidate for PDT, but also reveals the possibility of developing effective photosensitizers by complexing imidazole and piperazine into other hydrophobic photosensitizers.

## 1. Introduction

Photodynamic therapy (PDT) is a binary cancer therapy that relies on the selective uptake of a photosensitizer in tumor tissues, followed by generation of singlet oxygen and other cytotoxic species upon irradiation with light of an appropriate wavelength [1–2]. Singlet oxygen is considered to be the main cytotoxic species generated in PDT. It has a limited range of diffusion within tissues and it readily reacts with a variety of electron rich biomolecules such as unsaturated lipids, amino acids and DNA at the site of its generation. Although a number of studies have documented preferential uptake of photosensitizers in tumor tissues, the exact mechanism of PDT is not fully understood [3–5]. There are several possible approaches to explain preferential accumulation of photosensitizers in tumor cells relating to cellular specificity such as lower pH media and more low-density lipoprotein (LDL) receptors in malignant tissue than those in the normal tissue [6].

In order to find optimal photosensitizers, different compounds have been synthesized and examined including porphyrins, chlorins, phthalocyanines and purpurins [7–8]. Among them, chlorins represent the second generation of photosensitizers with promising physicochemical properties and high PDT efficiency [9–11].

Although one of the important prerequisites that photosensitizers should have is their amphiphilic property, most of chlorins are hydrophobic due to their hydrophobic macrocycle. If a photosenzitizer has too high lipophilicity, it has trouble to pass through blood vessels after intravenous injection. On the other hand, if a photosensitizer has too high hydrophilicity, it is difficult to penetrate cell membrane. Therefore it is important to compromise between hydrophilicity and lipophilicity. Introduction of hydrophilic groups imparts chlorin molecules with amphiphilic properties and, therefore, with good solubility in polar and non polar media. Such properties provide some chlorin derivatives with good tumor/tissue ratio, high tumor efficacy and short clearance time [12].

There have been several studies of protoporphyrin and chlorin e6 which are complexed with hydrophilic organic amine such as *N*-methyl-d-glucamine and arginine to improve their solubility in physiological solutions, while only few reports have been observed in the literature on pyropheophorbide-*a* derivatives complexed with hydrophilic organic amine used as a photosensitizer for PDT [13–14]. However, there is no research about salt formations between pyropheopheorbide-*a* and imidazole/piperazine. Therefore, we hypothesized that if we incorporated imidazole and piperazine into effective but hydrophobic photosensitizers, it may be possible to develop effective and amphiphilic photosensitizers without decreasing biological activities of parent chlorins in account of specific properties of imidazole and piperazine.

In this study, we aimed to develop effective photosensitizers which have good solubility in polar and non polar media while retaining the biological activities of the hydrophobic parent photosensitizers. Accordingly, we have designed two new salts of pyropheophorbide-*a* in which piperazine and imidazole groups, chosen as a hydrophilic organic amine, were non-covalently complexed to the propionic acid residue of pyropheophorbide-*a*. A comparative study of their photodynamic activities on A549 cancer cells was carried out in order to reveal influences of piperazine and imidazole moieties on biological activities of pyropheophorbide-*a.*

## 2. Results and Discussion

We have synthesized methyl pheophorbide-*a* (MPa) from *Spirulina pacifica* biomass by the procedure reported by Smith *et al.*, followed by further conversion into pyropheophorbide-*a* (PPa) by a two step chemical procedure including pyrolysis of MPa with 2,4,6-collidine and hydrolysis of methyl pyropheophorbide-*a* [15–17].

**Scheme 1:**
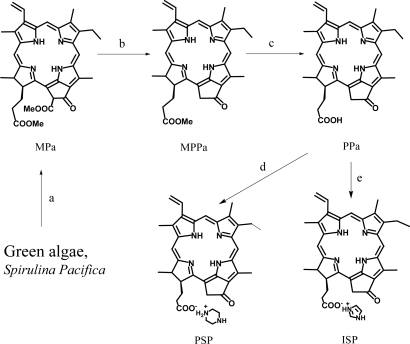
Synthetic pathways of piperazinium salt of pyropheophorbide-a (PSP) and imidazolium salt of pyropheophorbide-a (ISP). *Reagents*: a) 5% H_2_SO_4_, MeOH; b) Collidine; c) KOH, MeOH/THF; d) Piperazine/MeOH/ CH_2_Cl_2_ ; e) imidazole/MeOH/CH_2_Cl_2_.

The piperazinium and imidazolium salts of pyropheophorbide-a (PSP and ISP, respectively) were obtained by treating PPa with the organic free bases piperazine and imidazole, respectively. It is shown that formation of a salt bond between the carboxy group of the parent chlorin and the amine groups of piperazine and imidazole is a simple and effective method to obtain PPa salt-like complexes (Scheme 1). The ^1^H-NMR spectrum of PSP showed a broad singlet at 7.2 for the piperazine ring amine group protons, a singlet at 2.08 ppm for the piperazine ring imine group protons and also multiplets at 3.51–3.42 ppm for the protons of the four piperazine ring methylene groups, whereas ISP showed a broad singlet at 8.11 ppm for the imidazole ring imine group protons and singlets at 7.66 and 7.02 ppm, respectively, for the imidazole ring 2-position methine proton and the 4 and 5 position methine protons. Moreover, the chemical shifts for all protons of ISP appeared at slightly higher fields than those of PSP and Ppa. According to the ^1^H-NMR data, the formation of quaternary nitrogen on the imidazole and piperazine rings were confirmed by appearance of the characteristic peaks for imine and amine groups, respectively, on the rings.

### 2.1. Dark toxicity

**Figure 1:**
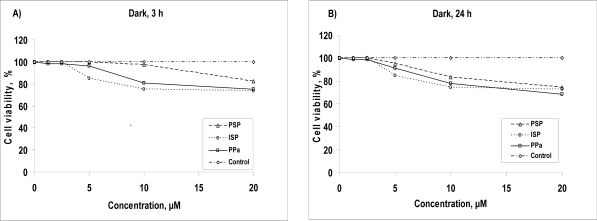
Dark toxicity of PSP (dash-“triangle”-dash), ISP (dot-“circle”-dot), starting material PPa (line-“square”-line) and control (dash-dot-“diamond”-dash-dot) toward A549 cells in dark without any irradiation for 3 h (A) and 24 h (B) incubation time.

The biological activity of PSP and ISP were evaluated in contrast to that of PPa in A549 human lung adenocarcinoma cells. An important prerequisite that a photosensitizer should have is a low dark toxicity when it is not irradiated. We therefore measured the dark toxicity of PSP and ISP by means of an MTT assay when cells treated by those compounds and untreated control cells kept in dark without any irradiation. After 3 h and 24 h of incubation in the dark, percentage of viable cells in each sample was measured by MTT assay (Figure 1).

At low concentrations, namely less than 3 μM, cells were not affected by the treatment with all compounds, whereas at concentrations more than 3 μM, all compounds exhibited a significant cytotoxic effect in A549 cells. Moreover dark toxicity of ISP was higher than those of PSP and PPa at concentrations more than 3 μM after 3h and 24 h incubation whereas the toxic effect induced by PSP was very lower than that of PPa at concentration more than 3 μM after 3 h and 24 h. To distinguish their dark toxicity effect quantitatively, we have evaluated the IC_90_ for each compound, which were 4.2 (ISP), 7.1 (PPa) 14.8 μM (PSP) after 3 h and, 4.1 (ISP), 5.3 (PPa) 7.2 μM (PSP) after 24 h, respectively. Determined IC_90_ values of compounds confirming that PSP exhibited drastically less toxic effect than both of ISP and PPa after 3h and 24 h incubation time.

### 2.2. Phototoxicity

**Figure 2:**
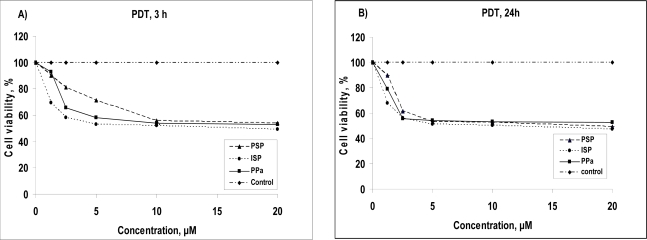
Phototoxicity of PSP (dash-“triangle”-dash), ISP (dot-“circle”-dot), starting material PPa (line-“square”-line) and control (dash-dot-“diamond”-dash-dot) toward A549 cells using 2 J cm^−2^ dose light for 3 h (A) and 24 h (B) incubation time after PDT.

We measured the phototoxicity of PSP, ISP and PPa at concentrations of 1.25, 2.5, 5, 10 and 20 μM in A549 cells when cells treated by those compounds and untreated control cells were irradiated with a 2 Jcm^−2^ light dose. The percentage of viable cells was evaluated by MTT assay at 3 h and 24 h incubation time after PDT with increasing concentrations of those compounds as shown in Figure 2.

Percentage of cell viability decreased with increasing concentration of agents for all photosensitizers. For 3 h incubation after PDT, ISP exhibited enhanced phototoxicity than starting material PPa at all concentrations whereas PSP showed decreased phototoxicity compared to PPa at all concentrations, except for 1.25 μM. In the 24 h incubation time case, the phototoxicity of ISP was higher than that of PSP at all concentrations, whereas PSP showed more phototoxicity than PPa at concentrations of more than 5 μM.

According to the dark and phototoxicity study of PSP and ISP comparing with PPa, PSP exhibited not only low dark toxicity but also high phototoxicity at concentrations of 1.25 μM, whereas ISP exhibited high phototoxicity for all concentrations, but showed slightly higher dark toxicity only for high concentrations. The increasements of dark and phototoxicity for ISP may be explained by specific properties of imidazole which is involved in anticancer and antibacterial medications [18–19].

**Figure 3:**
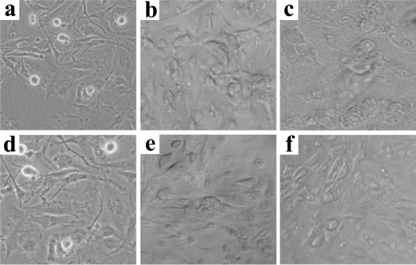
Optical image of morphological changes induced by PSP (b and c) and ISP (e and f) for 24 and 48 h respectively after irradiation compared with control (a and d) at 2.5×10^−6^ M concentration.

The phototoxicity of those compounds was also examined by monitoring cell death at 3 and 24 h incubation after photoirradiation under microscopic observation. Figure 3 shows the micrographs of A549 cells death at the concentration of 2.5 × 10-6 M and at different points of time after PDT. No cell death was observed without the agent, whereas cell death was observed at increased concentration of PSP and ISP. The leakage of the cytoplasm became more significant with time course and blebs were formed on the cell surface, indicating injury of the cell.

## 3. Conclusions

In summary, we have synthesized new amphiphilic photosensitizers which are soluble in water and non-polar media by complexing hydrophilic organic amines, namely imidazole and piperazine, with water-insoluble hydrophobic pyropeophorbide-*a*. Phototoxicity studies of those newly synthesized complexes have been performed to reveal the influence of the piperazine and imidazole moieties on the biological activities of pyropheophorbide-*a.* The results showed that complexation with piperazine and imidazole decreases the dark toxicity of pyropheophorbide-a without greatly decreasing phototoxicity and enhances its phototoxicity without greatly increasing dark toxicity, respectively. This work not only describes new, water-soluble salt complexes of pyropheophobide-*a* which retain the biological activities of the parent compound and could be effective candidates for PDT, but also shed a light on the development of promising amphiphilic photosensitizers by complexing other effective but hydrophobic photosensitizers with imidazole and piperazine. We are further investigating those amphiphilic salts for PDT to explore their aggregation in blood vessel after intravenous injection and their penetration and accumulation into cancer cells.

## 4. Experimental Section

### 4.1. Materials

Absorption spectra were measured in CH_2_Cl_2_ using a SCINCO S-3100 spectrophotometer. ^1^H-NMR spectra (300 MHz) were measured in CDCl_3_ solutions with TMS as internal standard using a UNITYplus-300 spectrometer (Busan Center of the Korean Basic Science Institute, Busan National University, Korea); chemical shifts are expressed in ppm relative to TMS (0.00). Silica gel 60A (230–400 mesh, Merck) was used for column chromatography. All reactions were monitored by thin-layer chromatography (TLC) using Merck 60 silica gel F_254_ precoated (0.2 mm thickness) glass-backed sheets. All chemicals, including imidazole, piperazine, MeOH, CH_2_Cl_2_, KOH, THF, SO_4_H_2_ and 1,3,5-collidine were analytical grade. Phosphate buffered saline (PBS, Sigma-Aldrich), a microscope (Olympus, CK40–32 PH, Japan), ELISA-reader (BioTek, Synergy HT, USA), trypsin-EDTA solution, and incubator (37°C, 5% CO_2_) were also used.

### 4.2. Synthesis of methyl pheophorbide-a

Dried *Spirulina Pacifica* alga (500 g) was refluxed in acetone (2 L) under nitrogen for two hours. The supernatant was filtered. The extraction and filtration process was repeated three times. The green filtrate was evaporated and treated with 5% SO_4_H_2_ in MeOH for 12.5 hours at room temperature in the dark under nitrogen. After that, reaction mixture was treated with CH_2_Cl_2_, rinsed with water, 10% aqueous NaCO3H solution, and again with water three times. The organic layer was separated and dried over anhydrous Na_2_SO_4_ and evaporated to dryness. The residue was purified by column chromatography on silica gel eluting with 2% acetone in CH_2_Cl_2_ and the product was re-crystallized from CH_2_Cl_2_-MeOH. Methyl pheophorbide-*a* was thus obtained in 0.4% yield.

### 4.3. Synthesis of the piperazinium salt of PPa (PSP)

To a solution of PPa (0.1 mmole) in MeOH/CH_2_Cl_2_ (3:1, 10 mL), a solution of piperazine (0.1 mmole) in MeOH/water (1:2, 20 mL) was added and the mixture stirred about 2 hours. The organic solvents were evaporated under vacuum and the resulting aqueous solution was filtered through a membrane (20 μm) and freeze dried to give the piperazinium salt of PPa. λ_max_(CH_2_Cl_2_) 667.3 nm; δ_H_ : 9.42 (1 H, s, H-5), 9.32 (1 H, s, H-20), 8.51 (1 H, s, H-10), 8.01 (1 H, m, H-3^1^), 7.20 (2H, s, H-NH_2_-pip), 6.28 and 6.16 (2H, d, H-3^2^), 5.30 and 5.09 (2 H, d, H-15^1^), 4.46 (1 H, q, H-18), 4.26 (1 H, d H-17), 3.75 (2H, q, H-8^1^), 3.60 (3 H, s, H-12), 3.51-3.41 (8H, m, H-CH_2_-pip), 3.36 (3 H, s, H-1^1^), 3.24 (3 H, s, H-7^1^), 2.7 (2 H, m, H-17^1^), 2.62 (2 H, m, H-17^2^), 2.08 (1H, br, NH-pip), 1.78 (3 H, d, H-18^1^), 1.67 (3 H, t, H-8^2^, *J* = 7.6 Hz, 7^2^ Me), 0.88 (1 H, br, H-NH); -1.72 (1H, br, H-NH).

### 4.4. Synthesis of the imidazolium salt of PPa (ISP)

To a solution of PPa (0.1 mmole) in MeOH/CH_2_Cl_2_ (3:1, 10 mL), a solution of imidazole (0.1 mmole) in MeOH/water (1:2, 20 mL) was added and the mixture stirred about 2 hours. The organic solvents were then evaporated under vacuum. The resulting aqueous solution was filtered through a membrane (20 μm) and freeze dried to give the imidazolium salt of PPa. λ_max_(CH_2_Cl_2_)/nm 667.1 nm; δ_h_ : 9.20 (1 H, s, H-5), 9.14 (1 H, s, H-20), 8.42 (1 H, s, H-10), 8.11 (2H, s, H-NH-imi), 8.01 (1 H, m, H-3^1^ ), 7.66 (1H, s, H^2^-imi), 7.03 (2H, s, H^4^ and H^5^ -imi), 6.19 and 6.08 (2H, d, H-3^2^), 5.28 and 5.03 (2 H, d, H-15^1^), 4.41 (1 H, q, H-18), 4.19 (1 H, d H-17), 3.72 (2H, q, H-8^1^), 3.45 (3 H, s, H-12), 3.27 (3 H, s, H-1^1^), 3.08 (3 H, s, H-7^1^), 2.64 (2 H, m, H-17^1^), 2.37 (2 H, m, H-17^2^), 1.73 (3 H, d, H-18^1^), 1.58 (3 H, t, H-8^2^, *J* = 7.6 Hz, 7^2^ Me), 0.87 (1 H, br, H-NH), -1.77 (1H, br, H-NH).

### 4.5. Cell culture and photoirradiation

The cell line tested was A549 (human lung carcinoma cell), which was obtained from the cell line bank at Seoul National University’s Cancer Research Center (Korea) and grown in RPMI-1640 medium (Sigma-Aldrich) with 10% fetal bovine serum, glutamine, penicillin and streptomycin at 37°C in a humidified atmosphere of 5% CO_2_ in air. The PDT was carried out using irradiation (BioSpec LED, Russia). The wavelength was set at an appropriate level depending on absorption maximum of the photosensitizer. Duration of the light irradiation, under PDT treatment, is calculated taking into account of the empirically found effective dose of light energy in J cm^−2^.

### 4.6. Morphological changes induced by PDT

Cells from each cell line were inoculated into a 96-well chamber slide at a volume of 100 μL (5 × 10^4^ cells/well) for stationary culture. Twenty-four h later, photosensitizer (2.5 μM, 100 μL/well) was then added. After a predetermined time, the photosensitizer solution was discarded and the culture was again washed (x 3) with physiological saline and medium added to a volume of 100 μL/well. The cultures were then subjected to LED irradiation at the distance of 20 cm for 10 min, and examined by optical microscopy 3 h and 24 h later to determine the morphologic changes induced, compared with the cultures not subjected to irradiation. The time course of the changes in survival rate after irradiation was observed.

### 4.7. Cell viability

Cells from each cell line were inoculated into a 96-well, flat-bottomed microplate at a volume of 100 μL (1 × 10^5^ cells/well) for stationary culture. Forty-eight hours later, the medium was removed, and the cultures were washed three times with physiologic saline. Amounts of photosensitizer solution (0.3, 0.6, 1.25, 2.5 and 5.0 μM) were then added at a volume of 100 μL/well, respectively. Twenty-four hours later, the photosensitizer solution was discarded, and the cultures were again washed three times with physiological saline and then medium was added to a volume of 100 μL/well. The cultures were then subjected to the irradiation (2 J cm^−2^) at the distance of 20 cm for 10 min, followed by an *3*-(*4*,*5*-dimethylthiazole-*2*-yl)-*2,5*-biphenyl tetrazolium bromide (MTT) assay to evaluate their sensitivity to PDT. For the MTT assay, MTT solution (10 μL) was added to each cell-culture well and cultured in the incubator for 3 h. Detergent solution (TACS™, Trevigen, 200 μL) was added to the culture, shaken for 10 min, and the absorbance was measured with an ELISA-reader at 570 nm. Measurements were performed 3 h and 24 h after the irradiation. Each group consisted of three wells.
